# Vectors and malaria transmission in deforested, rural communities in north-central Vietnam

**DOI:** 10.1186/1475-2875-9-259

**Published:** 2010-09-16

**Authors:** Cuong Do Manh, Nigel W Beebe, Van Nguyen Thi Van, Tao Le Quang, Chau Tran Lein, Dung Van Nguyen, Thanh Nguyen Xuan, Anh Le Ngoc, Robert D Cooper

**Affiliations:** 1 Entomology Department, Military Institute of Hygiene and Epidemiology, Hanoi, Vietnam; 2 School of Biological Sciences, University of Queensland, St Lucia, Queensland, 4072, Australia; 3 CSIRO Entomology, Long Pocket Laboratories, Indooroopilly, Queensland, 4068, Australia; 4 Military Preventive Medicine Centre, Ho Chi Minh City, Vietnam; 5 Australian Army Malaria Institute, Gallipoli Barracks, Enoggera, Queensland 4051, Australia

## Abstract

**Background:**

Malaria is still prevalent in rural communities of central Vietnam even though, due to deforestation, the primary vector *Anopheles dirus *is uncommon. In these situations little is known about the secondary vectors which are responsible for maintaining transmission. Basic information on the identification of the species in these rural communities is required so that transmission parameters, such as ecology, behaviour and vectorial status can be assigned to the appropriate species.

**Methods:**

In two rural villages - Khe Ngang and Hang Chuon - in Truong Xuan Commune, Quang Binh Province, north central Vietnam, a series of longitudinal entomological surveys were conducted during the wet and dry seasons from 2003 - 2007. In these surveys anopheline mosquitoes were collected in human landing catches, paired human and animal bait collections, and from larval surveys. Specimens belonging to species complexes were identified by PCR and sequence analysis, incrimination of vectors was by detection of circumsporozoite protein using an enzyme-linked immunosorbent assay.

**Results:**

Over 80% of the anopheline fauna was made up of *Anopheles sinensis*, *Anopheles aconitus*, *Anopheles harrisoni*, *Anopheles maculatus*, *Anopheles sawadwongporni*, and *Anopheles philippinensis*. PCR and sequence analysis resolved identification issues in the Funestus Group, Maculatus Group, Hyrcanus Group and Dirus Complex. Most species were zoophilic and while all species could be collected biting humans significantly higher densities were attracted to cattle and buffalo. *Anopheles dirus *was the most anthropophilic species but was uncommon making up only 1.24% of all anophelines collected. *Anopheles sinensis*, *An. aconitus*, *An. harrisoni*, *An. maculatus*, *An. sawadwongporni, Anopheles peditaeniatus *and *An. philippinensis *were all found positive for circumsporozoite protein. Heterogeneity in oviposition site preference between species enabled vector densities to be high in both the wet and dry seasons allowing for year round transmission.

**Conclusions:**

In rural communities in north central Vietnam, malaria transmission was maintained by a number of anopheline species which though collected feeding on humans were predominantly zoophilic, this behaviour allows for low level but persistent malaria transmission. The important animal baits - cattle and buffalo - were kept in the village and barrier spraying around these animals may be more effective at reducing vector densities and longevity than the currently used indoor residual spraying.

## Background

Malaria is endemic in many parts of Vietnam and up until the 1990s was a major public health problem. Since then a concerted effort by the government through the distribution of insecticide-treated nets and wide spread availability of artesunate treatment has significantly reduced transmission [1-3]. Much of the morbidity and mortality that now remains is associated with forest malaria in the central highland regions of the country where *Anopheles dirus*, an efficient vector of malaria, is common [4-6].

While the control of *An. dirus *and forest malaria is a significant problem for Vietnam there are still many rural areas throughout the country where, though the land has been cleared for cultivation and *An. dirus *is uncommon, malaria still persists. In these deforested areas, in the absence of *An. dirus*, other species - *Anopheles minimus *s.l., *Anopheles aconitus*, *Anopheles maculatus *s.l., and *Anopheles sinensis *- have been considered responsible for malaria transmission. However there is little published data confirming the identification of these species or their distribution and thus there is little known about their ecology, behavior and the vectorial status.

Acquiring the relevant epidemiological information pertaining to these species and confirmation of their role in transmission has been impeded by the presence of cryptic species within many of the suspected vector taxa. This has recently been resolved for a number of these complexes and reliable molecular techniques are now available allowing the accurate identification of the various complex members. Molecular based techniques have been developed for the identification of members of the *An. dirus *[7,8], *An. minimus *[9], *An. maculatus *[10], *Anopheles annularis *[11], and *Anopheles sundaicus *[12], groups and complexes and thus it is now possible to conduct field studies to determine their distribution, ecology, behavior, and role in malaria transmission.

Truong Xuan Commune in Quang Binh Province (north central Vietnam) is typical of many rural communities in Vietnam where the forest surrounding the villages has been cleared for timber and cultivation, the primary malaria vector - *An. dirus *- is uncommon yet a low level of malaria persists throughout the year. Transmission occurs within the village, and while some forest still remains on the surrounding hills no agricultural activities occur there. These forests are only visited for the purposes of timber cutting, hunting, and food gathering; these transient activities do not allow the existence of forest malaria [6]. In Truong Xuan little is known about the vectors of malaria, to resolve this, entomological surveys were conducted in two villages in the Commune over the period 2003-2007; the findings of these surveys are reported here.

## Methods

### Study site

Truong Xuan Commune in Quang Binh Province is located 17° 17'N and 106° 37'E, and is approximately 500 km south of Hanoi. The commune consists of several villages, including the two study sites: Hang Chuon village (26 houses, population approximately 100) and Khe Ngang village (48 houses, population approximately 200). These two villages are 15 km inland and lie in a river valley (17 m above sea level) surrounded by limestone mountains. Hang Chuon and Khe Ngang are 2 km apart and separated by mountains, but connected by a narrow pass. A river flows through this pass and through both villages. The river is fed by numerous small streams which are slow flowing even in the wet season but are reduced to small interconnected pools in the dry season. The climate of the region is tropical monsoon with distinct wet and dry seasons. The area receives about 1,960 mm of rain per annum (median for 1993-2002) with 70% occurring during the wet season from August to December; rain during this period can be intense and cause local flooding. Mean temperatures (mean of max/min over 10 yr) range from 17.1°C in February to 29.7°C in July.

Hang Chuon is slightly undulating, around both villages the original forest has been cleared for cultivation, which consists mainly of cassava, corn, and melons; rice is grown around Khe Ngang. A variety of blood sources exist for mosquitoes and while all households have dogs and chickens the larger blood sources are humans, cattle, and buffalo. Cattle and buffalo free range during the day, when not used for work, but at night are penned or tethered near the owner's house (within 15 m). Most houses in Khe Ngang are rendered brick while in Hang Chuon, which appears less affluent, many of the houses are of a more traditional style, raised off the ground (1-2 m) and of very loose or open constructions with walls of woven bamboo or cane matting. Insecticide treated bed nets are provided by the government and indoor residual spraying with permethrin has been carried out once a year since 2002. The villages are serviced by the Commune Health Station which is about 6 km from Khe Ngang and 8 km from Hang Chuon. Malaria diagnosis is by blood-slide microscopy and free treatment - a seven day course of artesunate - is provided, though compliance is not observed. In the villages health workers also provide treatment for symptomatic cases.

### Incidence of malaria

The incidence of malaria was determined by passive case detection through the Commune Health Station. Diagnosis was by microscopy, based on examination of 100 thick film fields of a Giemsa-stained slide (4% and stained for 45 minutes). Positive slides were then read against 200 white blood cells (presuming 8-10 WBC/thick film field) to determine species and parasite density (parasite density was scored as: +, ++, +++, ++++). The clinic maintained comprehensive records of the name, age, sex, village, slide positivity and species of *Plasmodium*.

There is no transport system throughout the commune and while push bike and motor bike ownership is not uncommon, a visit to the Health Station could involve a walk of up to 20 km. The Commune Health Station is supported by village health workers who operate at the village level and who diagnoses and treat malaria cases based on symptoms.

### Anopheline collections

Four collection methods were employed: human landing catches (HCL); paired collections off humans and buffalo; simultaneous collections off humans, cattle, and buffalo; and larval collections. These were performed as follows.

Human landing collections were made in Khe Ngang village during the wet seasons (September - October) of 2003 and 2004 and similar HLC were carried out in Hang Chuon village during the wet seasons of 2004 and 2005. These HLC were made by four collectors in each village, all collectors worked outdoors. Each collector caught all anophelines landing on the lower legs and feet for 50 minutes each hour from 6 pm - 6 am. All catches were held in cups labelled for the hour; the mosquitoes were killed by freezing and identified the following morning.

At Khe Ngang collections were made over 27 nights over the two wet season; rain interrupted collections at Hang Chuon and the number of collection nights varied from 14 - 27 nights over two wet seasons.

Paired anopheline collections off buffalo and human baits were performed at Khe Ngang village for 10 nights over the wet seasons (September - October) of 2004 and 2005. Collections were made hourly from 6 pm - 6 am, with one collector collecting off two tethered buffalo and by one collector sitting 15 m away collecting off himself. All collections were for 50 min each hour and, for the human bait, performed as described above for the HLC. Collections from the buffalo were made by searching the surrounding vegetation (within 2 m of the animals) for resting anophelines. All mosquitoes were held separately by hour and bait type, killed by freezing and identified the following morning.

Simultaneous anopheline collections were made off human, buffalo, and cattle baits at Hang Chuon village for 14 nights during the wet season of 2006 and for 14 nights during the dry season (April - May) of 2007. The collections were made by six collectors, two collecting off cattle, two collecting off buffalo, and two collecting off themselves; the collections were made for two hours from 8 pm - 10 pm. Anophelines were collected and processed as described above.

A search for anopheline larvae was made within 1-2 km of Khe Ngang and Hang Chuon villages during the wet and dry seasons 2005-2007. All larvae collected were reared to adults and identified. A description of the oviposition site was recorded.

All specimens from the adult and larval collections were held frozen (-20°C) in the field, transported back to the laboratory on dry ice (-70°C) and stored at this temperature until being analysed for the presence of circumsporozoite protein or by PCR for species identification.

### Species identification

All specimens were identified in the field using the national key - Identification Key for *Anopheles *in Vietnam 1987 - prepared by the Institute of Malaria, Parasitology and Entomology, Hanoi.

Specimens belonging to the Hyrcanus Group, Funestus Group, Maculatus Complex, and Dirus complex were further analysed using the Internal Transcribed Spacer region 2 (ITS2) of the ribosomal DNA by either polymerase chain reaction - restriction fragment length polymorphism (PCR-RFLP) or allele-specific PCR analyses as well as DNA sequencing. For RFLP analysis, DNA was extracted, the ITS2 region amplified, digested with an appropriate restriction enzyme and the RFLP generated separated and visualized using previously described methods [13,14]. Additional methods were used to resolve the identification of the members of the Dirus Complex [8], the Funestus Group [9], and the Maculatus Group [10]. All PCR-based methods for species identification were validated by DNA sequencing individuals representing each species-specific product and comparing the ITS2 sequences with those listed in GenBank.

### Vector incrimination

The head and prothorax of specimens collected off human, cattle, and buffalo baits were processed for the presence of circumsporozoite protein of *Plasmodium falciparum *and *Plasmodium vivax *using an enzyme linked immunoabsorbent assay (ELISA) and the protocol of Dr Robert Wirtz (Centers for Disease Control and Prevention, MS F42, Atlanta, GA 30341-3717, USA). Specimens were considered positive if the absorbance value recorded was twice that of the average of the negative controls and all positive samples were rerun for confirmation.

## Results

### Malaria incidence in Truong Xuan

Between 2002 and 2006, 509 malaria cases reported to the Health Station - an average of 102 per annum and indicating an annual parasite rate of 6.9% (102/1467) for the Commune. This though was likely to be an underestimate of the malaria situation as some people self medicated or were treated in the village and others who were asymptomatic or who only had mild symptoms would not travel the distance to the Commune Health Station; all these cases go unreported. Malaria does not occur evenly throughout the commune and of the several villages that make up Truong Xuan Commune the range of malaria cases was 4 to 136. Hang Chuon and Khe Ngang, two of the more remote villages, had the highest incidences of transmission making up 26.7% (136/509) and 14.1% (72/509) of the cases recorded respectively. In these two villages adult females and children (< 9 yr) accounted for 60.3% of the malaria indicating that transmission was occurring in the villages as these inhabitants stay in the village at night.

**Figure 1:**
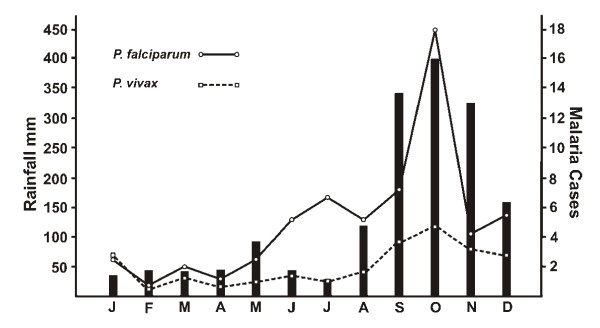
**Monthly rain fall data (mean of 10 years) and the mean monthly incidence of malaria (2003-2007) for Truong Xuan Commune**.

Of the 509 cases 71.9% were *P. falciparum *and 28.1% *P. vivax*; malaria transmission occurred all year round but peaked during the wet season in the month of October (Figure 1).

### Anopheline identification

Of the mosquitoes collected many were identified as belonging to complexes or groups of species which were difficult to separate using morphological characters provided in the national key. For the anophelines at Truong Xuan Commune molecular analysis redefined species composition, condensing some groups and revealing cryptic species in others.

From the collections made in Khe Ngang and Hang Chuon the following members of the Hyrcanus Group were identified by morphology: *An. sinensis*, *Anopheles peditaeniatus*, *Anopheles crawfordi*, *Anopheles nigerrimus*, *Anopheles argyropus*, *Anopheles lesteri*, and *Anopheles nitidus *(= *Anopheles indiensis *by the national key). Using the amplified ribosomal ITS2 region and digesting with the restriction enzyme Dde I, diagnostic restriction fragments length polymorphisms (RFLP) were produced that resolved these eight species to *An. sinensis*, *An. peditaeniatus*, and *An. crawfordi *(Table 1). The ITS2 regions of individuals representing each of these species were sequenced and the sequences matched to those on GenBank confirming the species as indicated. In Khe Ngang the proportion of *An. sinensis*, *An. peditaeniatus*, and *An. crawfordi *was 92.0%, 7.6% and 0.4% respectively (n = 1,648), in Hang Chuon: 56.2%, 43.8% and 0% respectively (n = 130).

**Table 1:** Resolution of identification by PCR-RFLP for members of the Hyrcanus Group collected in Khe Ngang village.

	Identification by:			
	
Species	Morphology	PCR-RFLP		
		
		*An. sinensis*	*An. peditaeniatus*	*An. crawfordi*
*An. sinensis*	1379	1353	24	2
*An. peditaeniatus*	104	40	64	0
*An. nigerrimus*	75	44	29	2
*An. crawfordi*	23	19	1	3
*An. nitidus*	34	33	1	0
*An. argyropus*	11	7	4	0
*An. lesteri*	22	20	2	0
Total %	1648	1516 92.0%	125 7.6%	7 0.4%

Three members of the Funestus Group were identified in the collections: *An. minimus *s.l. of the Minimus Complex, *An. aconitus*, and *Anopheles jeyporiensis*. Morphologically the white scaling on the apical half of the proboscis reliably identified *An. aconitus*. Following molecular analysis the majority of *An. minimus *s.l. were found to be *Anopheles harrisoni *with only a small number of *An. minimus*; of the 20 *An. jeyporiensis *collected only one belonged to this species, the remainder were *An. harrisoni *(Table 2).

**Table 2:** Resolution of identification by PCR-RFLP for members of the Funestus Group collected in Hang Chuon village.

	Identification by:				
	
Species		PCR			
		
	Morphology	***An. minimus***	***An. harrisoni***	***An. aconitus***	***An. jeyporiensis***
*An. minimus *s.l.	1034	15	1017	1	1
*An. jeyporiensis*	20	0	19	0	1
*An. aconitus*	1152	1	1	1150	0

In the national key the only member of the Maculatus Group described in Vietnam is *An. maculatus*. Of 2,121 specimens of this species collected in Hang Chuon and Khe Ngang and analysed by PCR-RFLP 47.2% were found to be *An. maculatus *and 52.8% were *An. sawadwongporni*. The restriction enzyme Hsp92 II was found to produce diagnostic RFLP for separating these two species, this outcome was confirmed by sequencing and matching to existing sequences in GenBank.

Of 63 *An. dirus *s.l. specimens analysed by PCR-RFLP all were *An. dirus *(formerly *An. dirus *A); seven specimens identified morphologically as *Anopheles takasagoensis *were also found to be *An. dirus*.

### Species composition and host preference

From 2003 to 2007, 10,078 anophelines were collected off human and animal baits. Using morphology and molecular analysis 21 species were identified from this material (Table 3). Six species: *An. sinensis*, *An. aconitus*, *An. harrisoni*, *An. maculatus*, *An. sawadwongporni *and *An. philippinensis *were the most common and accounted for 80.5% of all the specimens collected. While all species, except for some of the less common ones (< 10 specimens collected), were collected from humans the majority (80.94%) of specimens were collected off non-human (cattle and buffalo) baits (Table 3). Of the species common to both villages, those in Hang Chuon appeared to be more zoophilic than those in Khe Ngang (Tables 4 and 5). Only *An. dirus *showed anthropophilic tendencies, though the numbers of this species collected in human and animal bait studies was low. *Anopheles sawadwongporni*, *An. sinensis*, and *An. maculatus *were the least zoophilic of the other species while *An. philippinensis*, *An. vagus*, *An. kochi*, *An. nivipes*, and *An. harrisoni *were strongly zoophilic (Tables 4 and 5). In Hang Chuon village a comparison between the host attractiveness of buffalo or cattle indicated that most species preferred to feed off cattle or had no preference at all (Table 5). Only *An. philippinensis *appeared to have a preference for buffalo but this was not statically significant (χ^2 ^= 3.38, P = 0.1).

**Table 3:** *Anopheles *species and numbers collected off human and animal (buffalo and cow) baits during 2003-2007 in Khe Ngang and Hang Chuon villages.

Species (abbreviation)	Bait		Total	%
			
	Human	Animal		
*An. sinensis *(sin)	709	1792	2501	25.01
*An. aconitus *(aco)	171	1266	1437	14.37
*An. harrisoni *(har)	71	938	1009	10.09
*An. maculatus *(mac)	285	877	1162	11.62
*An. sawadwongporni *(saw)	424	764	1188	11.88
*An. philippinensis *(phil)	72	749	821	8.21
*An. vagus *(vag)	8	578	586	5.86
*An. annularis *(ann)	37	418	455	4.55
*An. kochi *(koc)	4	326	330	3.30
*An. peditaeniatus *(ped)	11	192	203	2.03
*An. nivipes *(niv)	5	166	171	1.71
*An. dirus *(dir)	111	13	124	1.24
*An. barbirostris *(bar)	2	36	38	0.38
*An. minimus *(min)	5	14	19	0.19
*An. umbrosus *(umb)	0	8	8	0.08
*An. crawfordi *(cra)	0	8	8	0.08
*An. separatus *(sep)	0	7	7	0.07
*An. jamesi *(jam)	2	2	4	0.04
*An. tessellatus *(tes)	2	2	4	0.04
*An. jeyporiensis *(jey)	0	2	2	0.02
*An. gigas *(gig)	1	0	1	0.01
Totals	1920	8158	10078	100

**Table 4:** *Anopheles *species and numbers collected simultaneously off human and buffalo baits in Khe Ngang village during the wet seasons of 2004 and 2005.

	Bait type		
	
Species	Human	Buffalo	AI
*An. sawadwongporni*	9	30	0.300
*An. maculatus*	4	48	0.083
*An. harrisoni*	2	60	0.033
*An. sinensis*	60	1852	0.024
*An. aconitus*	26	1163	0.022
*An. vagus*	2	110	0.018
*An. annularis*	6	349	0.017
*An. kochi*	2	126	0.015
*An. philippinensis*	6	524	0.011
*An. dirus*	4	0	-
*An. nivipes*	0	8	-
*An. peditaeniatus*	0	41	-
*An. barbirostris*	0	24	-
*An. crawfordi*	0	8	-

Collections were made from 6pm - 6am over 10 nights. Note AI is the anthropophilic index (human bait/animal bait).

**Table 5:** *Anopheles *species and numbers collected simultaneously off human, cattle and buffalo baits in Hang Chuon village.

	Bait type				
	
Species	Human	Cattle	Buffalo	**χ**^**2**^	AI
*An. dirus*	9	8	5	0.346	0.692
*An. sawadwongporni*	33	528	206	70.63**	0.045
*An. sinensis*	1	28	17	1.34	0.022
*An. peditaeniatus*	1	29	19	1.04	0.021
*An. maculatus*	14	504	327	18.85**	0.017
*An. philippinensis*	1	93	132	3.38	0.004
*An. harrisoni*	2	503	375	9.33*	0.002
*An. aconitus*	0	62	41	2.14	-
*An. annularis*	0	36	33	0.065	-
*An. barbirostris*	0	9	3	-	-
*An. jamesi*	0	1	1	-	-
*An. kochi*	0	129	71	8.41*	-
*An. minimus*	0	6	3	-	-
*An. nivipes*	0	4	4	-	-
*An. tessellatus*	0	2	0	-	-
*An. umbrosus*	0	0	3	-	-
*An. vagus*	0	300	168	18.61**	-

Collections were made from 8pm - 10pm over 28 nights (14 nights during the wet season of 2006 and 14 nights during the dry season of 2007). Note χ^2 ^is a comparison of the attractiveness of cattle and buffalo for the various species of anophelines, with * significant at P < 0.05 and **significant at P < 0.001.

### Species composition and heterogeneity between villages

Hang Chuon and Khe Ngang are only 2 km apart and there was no one species unique to either village. However abundance of the various species did vary. In Khe Ngang *An. sinensis*, *An. aconitus*, and *An. philippinensis *were the three most common species collected in HLC, while at Hang Chuon *An. sawadwongporni*, *An. maculatus*, and *An. dirus *were the three most common species collected in HLC (Tables 6 and 7).

**Table 6:** Human landing catch rates for *Anopheles *species in Khe Ngang village during the wet seasons (September - October) of 2003 and 2004.

	Khe Ngang
	
Species	landing/person/night
*An. sinensis*	12.42
*An. aconitus*	1.91
*An. philippinensis*	1.01
*An. annularis*	0.58
*An. sawadwongporni*	0.34
*An. harrisoni*	0.27
*An. maculatus*	0.22
*An. vagus*	0.20
*An. dirus*	0.18
*An. kochi*	0.07
*An. nivipes*	0.07
*An. tessellatus*	0.04
*An. barbirostris*	0.02
*An. minimus*	0
*An. peditaeniatus*	0
All species	13.55

**Table 7:** Human landing catch rates for *Anopheles *species in Hang Chuon village during the wet seasons (September - October) of 2004 and 2005.

	Hang Chuon
	
Species	landing/person/night
*An. sawadwongporni*	4.68
*An. maculatus*	3.06
*An. dirus*	1.29
*An. aconitus*	0.69
*An. harrisoni*	0.60
*An. philippinensis*	0.20
*An. sinensis*	0.19
*An. peditaeniatus*	0.13
*An. annularis*	0.06
*An. minimus*	0.06
*An. vagus*	0.06
*An. barbirostris*	0.01
*An. nivipes*	0.01
*An. kochi*	0
*An. tessellatus*	0
All species	10.06

### Seasonality

In Hang Chuon, for some species, abundance differed depending upon the season. Most species were more common in the wet season, notably *An. sawadwongporni*, *An. philippinensis*, and *An. vagus*. Whereas, *An. harrisoni*, *An. sinensis*, and to some extent *An. aconitus *were more prolific in the dry season. However, *Anopheles maculatus*, *An. dirus*, and *An. peditaeniatus *numbers appeared to be unaffected by rainfall patterns (Table 8).

**Table 8:** Comparison of *Anopheles *species and numbers collected off human, cattle, and buffalo baits during the wet season and dry season.

Species	Collected in dry season	Collected in wet season	**χ**^**2**^
*An. aconitus*	65	38	3.539
*An. annularis*	12	57	14.674**
*An. barbirostris*	11	1	4.167*
*An dirus*	10	12	0.090
*An. kochi*	79	121	4.410*
*An. maculatus*	415	416	0.0006
*An. sawadwongporni*	83	651	219.77**
*An. harrisoni*	759	121	232.53**
*An. minimus*	7	2	-
*An. nivipes*	5	3	-
*An. peditaeniatus*	22	27	0.225
*An. philippinensis*	29	197	62.442**
*An. sinensis*	41	5	14.086**
*An. tessellatus*	1	1	-
*An. umbrosus*	3	0	-
*An. vagus*	78	390	104.00**
Total	1620	2042	

Collections were made over 14 nights in the wet season of 2006 and 14 nights in the dry season of 2007 in Hang Chuon village. Note χ^2 ^is a comparison of wet and dry season collections for each species where * indicates significance at P < 0.05 and ** a significance at P < 0.001

### Feeding behaviour

The night feeding pattern for the common species coming to human and buffalo baits are shown in Tables 9 and 10 and human landing catches throughout the night are shown in Table 11 for the common species in Khe Ngang and Table 12 for those in Hang Chuon. Feeding patterns off humans and animals appeared similar for the same species. Some species - *An. sinensis*, *An. annularis*, *An. aconitus*, *An. philippinensis*, *An. nivipes*, and *An. vagus*- fed throughout the night commencing at sunset and rising to a peak at about midnight (11 pm-1 am) and then remaining high until just before dawn. While others - *An. maculatus*, *An. sawadwongporni, An. dirus*, and to some extent *An. harrisoni*, tended to seek a host early in the evening with human landing catches peaking between 6 pm - 9 pm. All mosquitoes left the vicinity of the animal bait immediately at first light (5.30 am - 5.45 am) and no human landing occurred after this time. In the collections off cattle and buffalo it was noted that anophelines rested before and after feeding on the vegetation surrounding these hosts.

**Table 9:** Anopheline collections off human baits in Khe Ngang village during the wet seasons (September-October) of 2004 and 2005.

	Species collected landing on human bait									
	
Hour	sin	ann	aco	phil	mac	saw	har	koc	vag	dir
6-7	4	1	0	0	1	0	1	0	1	1
7-8	5	1	2	0	0	1	0	0	0	1
8-9	2	0	3	1	1	0	0	0	0	0
9-10	4	1	4	1	0	1	0	0	0	0
10-11	9	1	7	0	1	0	0	0	0	0
11-12	5	0	2	0	0	0	0	0	0	0
12-1	8	0	2	2	0	0	0	2	0	1
1-2	9	1	2	0	1	3	0	0	1	1
2-3	5	1	3	1	0	2	0	0	0	0
3-4	5	0	1	0	0	1	1	0	0	0
4-5	1	0	0	1	0	1	0	0	0	0
5-6	3	0	0	0	0	0	0	0	0	0
Totals	60	6	26	6	4	9	2	2	2	4

Collections were conducted hourly from 6pm to 6am over 10 nights collections were made simultaneously with collections off buffalo bait see Table 10. Total number of specimens collected = 121; of the 60 *An. sinensis *collected all were *An. sinensis*.

**Table 10:** Anopheline collections off buffalo baits in Khe Ngang village during the wet seasons (September-October) of 2004 and 2005.

	Species collected from buffalo bait										
	
Hour	sin	ann	aco	phil	niv	mac	saw	har	bar	koc	vag
6-7	108	6	19	10	4	4	0	4	6	6	1
7-8	111	32	34	35	11	6	5	6	3	16	8
8-9	137	16	44	32	28	8	3	5	2	6	7
9-10	135	20	61	61	15	8	4	1	2	4	24
10-11	148	27	135	50	12	3	3	4	3	18	9
11-12	188	50	141	58	2	4	4	9	3	15	12
12-1	183	34	140	66	13	4	3	6	2	9	11
1-2	158	47	143	50	9	3	2	8	1	20	8
2-3	199	46	120	39	13	2	2	3	0	15	7
3-4	188	32	107	43	15	0	2	5	1	6	6
4-5	218	28	157	44	32	2	2	7	1	7	13
5-6	126	11	62	36	4	2	0	2	0	4	4
Totals	1899	349	1163	524	158	46	30	60	24	126	110

Collections were conducted hourly from 6pm to 6am over 10 nights collections were made simultaneously with collections off human bait see Table 9. Total number of specimens collected = 4,489; of the 1,899 An. sinensis collected 1,852 were *An. sinensis*, 41 *An. peditaeniatus *and 6 *An. crawfordi*

**Table 11:** Night landing pattern for six *Anopheles *species collected off human bait in Khe Ngang village in the wet seasons (September- October) of 2003-2004.

		Khe Ngang landing/person/hour	
		
Hour	No. of nights	*An. sinensis *n = 648	*An. aconitus *n = 145
6-7	27	0.46	0.12
7-8	27	0.55	0.23
8-9	27	0.55	0.25
9-10	27	0.96	0.37
10-11	27	1.7	0.47
11-12	27	1.4	0.19
12-1	27	1.2	0.21
1-2	27	1.2	0.30
2-3	27	1.2	0.21
3-4	27	1.6	0.14
4-5	27	1.1	0.09
5-6	27	0.5	0.10
landing/person/night		12.42	2.68

**Table 12:** Night landing pattern for six *Anopheles *species collected off human bait in Hang Chuon village in the wet seasons (September- October) of 2004-2005.

		Hang Chuon landing/person/hour			
		
Hour	No. of Nights	*An. dirus *n = 98	*An. harrisoni *n = 67	*An. maculatus *n = 267	*An. sawadwongporni *n = 391
6-7	27	0.115	0.17	1.03	0.95
7-8	27	0.145	0.13	0.42	0.63
8-9	27	0.20	0.07	0.27	0.44
9-10	19	0.10	0.04	0.18	0.38
10-11	19	0.15	0.10	0.08	0.32
11-12	19	0.04	0.040	0.03	0.26
12-1	14	0.12	0	0.11	0.34
1-2	14	0.12	0.09	0.20	0.21
2-3	14	0.13	0.06	0.11	0.20
3-4	14	0.12	0.04	0.09	0.32
4-5	14	0.03	0.11	0.13	0.30
5-6	14	0.07	0.11	0.41	0.43
landing/person/night		1.29	0.93	3.06	4.68

### Larval habitats

The preference for, and availability of, particular habitats can account for differences in the distribution, abundance, and seasonality of individual species. Table 13 lists the oviposition sites for the most common species in Khe Ngang and Hang Chuon. The main difference between these two villages, with regards to larval habitats, was the presence of rice fields around Khe Ngang and the greater extent of riparian habitat around Hang Chuon. In Truong Xuan Commune rice is planted in the wet season during October - November and harvested in May, but pools remain in rice fields long after harvest. *Anopheles sinensis *had a significant associated with rice growing (χ^2 ^= 22.5, P < 0.001), which would explain its abundance around Khe Ngang. Rice fields are flooded in October but contain pools of water long after the rice is harvested in May, *An. sinensis *will use flooded rice fields but prefers the pools that were left post-harvest; thus *An. sinensis *can occur year round but was more common in the dry season than the wet season. *Anopheles harrisoni*, *An. maculatus*, and *An. sawadwongporni *all had a significant association with pools in stream and river beds (χ^2 ^> 38.4, P < 0.001) and were more abundant in Hang Chuon, which has a more extensive net work of rivers and streams. Dry season conditions seem to favour the abundance of *An. harrisoni *(Table 8), of the 18 larval sites located for this species in Hang Chuon in the dry season of 2006 only one was found in the following wet season. However with *An. sawadwongporni *there appeared to be no correlation between seasonal abundance and the availability of larval habitats, this species was more prolific in the wet season but in 2006 nearly the same number of larval sites were found for this species in the dry season (n = 14) as in the wet season (n = 17). Of 42 larval habitats for *An. maculatus *and *An. sawadwongporni *located at Hang Chuon in 2006, both species co-habited in 13 sites. *Anopheles vagus*, a common and adaptable species, utilised a wide variety of ground pools and was found in all habitats. *Anopheles dirus *larvae were found in only one site - a small pool in a jungle stream - this collection was made in the dry season of 2004 but was not found there again at this site over the next three years.

**Table 13:** Larval habitats for the common anopheline species collected in Khe Ngang and Hang Chuon during 2004 - 2007.

	Larval habitat used (%)			
	
Species (Number of habitats located)	Rice fields	Ground pools	Riparian	Flooded grassland
*An. vagus *(42)	8 (19.1)	26 (61.9)	6 (14.3)	2 (4.8)
*An. sinensis *(40)	25 (62.5)	12 (30.0)	1 (2.5)	2 (5.0)
*An. maculatus *(31)	2 (6.4)	3 (9.7)	25 (80.6)	1 (3.2)
*An. sawadwongporni *(30)	0	0	29 (96.7)	1 (3.3)
*An. harrisoni *(23)	0	1 (4.3)	21 (91.3)	1 (4.3)
*An. annularis *(19)	7 (36.8)	8 (42.0)	1 (5.3)	3 (15.8)
*An. aconitus *(19)	6 (31.6)	8 (42.0)	3 (15.8)	2 (10.5)
*An. philippinensis *(15)	5 (33.3)	5 (33.3)	0	5 (33.3)
*An. barbirostris *(10)	2 (20.0)	2 (20.0)	6 (60.0)	0
*An. peditaeniatus *(7)	4 (57.1)	3 (42.9)	0	0
*An. kochi *(8)	7 (87.5)	1 (12.5)	0	0
*An. nivipes *(5)	2 (40.0)	1 (20.0)	0	2 (40.0)
*An. dirus *(1)	0	0	1 (100)	0

Rice fields includes those flooded for cultivation and pools post-harvest, also included are associated pools used for regulating water in rice fields; ground pools includes buffalo wallows, borrow pits, natural depressions, fish ponds, and pools in drains; riparian habitat includes pools associated with rivers and streams, pools along the margins of rivers and streams, turbulence pits, pools in drying streams, rock pools; and flooded grassland includes water meadows inundated with water from irrigation runoff, flood water runoff, overflow from rivers and streams.

### Vector status

Specimens of all the common species collected off human and animal baits were assayed for CS protein and the findings presented in Table 14. Several species were found positive for CS protein; of these *An. sinensis *was the main malaria vector in Khe Ngang, where it was most common, while *An. harrisoni*, *An. maculatus *and *An. sawadwongporni *were the main vectors in Hang Chuon.

**Table 14:** *Anopheles *species tested positive for circumsporozoite protein.

Species	No. tested	No. positive (SR)	Village	Collection method and malaria parasite species
*An. sinensis*	1442	12 (0.01)	Khe Ngang	human bait: Pf × 4, Pv 247 × 2, Pv 210 × 1 animal bait: Pf × 1, Pv 247 × 3, Pv 210 × 1
*An. harrisoni*	997	3 (0.003)	Hang Chuon	animal bait: Pf × 2 human bait: Pf × 1
*An. maculatus*	1112	2 (0.002)	Hang Chuon	human bait: Pf × 1 animal bait: Pf × 1
*An. sawadwongporni*	1120	1 (0.001)	Hang Chuon	human bait: Pf
*An. aconitus*	1539	1 (0.0006)	Khe Ngang	human bait: Pf
*An. peditaeniatus*	131	1 (0.008)	Khe Ngang	animal bait: Pv 247
*An. philippinensis*	658	1 (0.002)	Khe Ngang	human bait: Pv 247
*An. vagus*	586	0		
*An. annularis*	366	0		
*An. kochi*	136	0		
*An. dirus*	123	0		
*An. minimus*	14	0		

Where Pf = *Plasmodium falciparum *and Pv = *Plasmodium vivax *(210 and 247 variant); mosquitoes were collected over the period 2004-2007. Where SR = sporozoite rate.

## Discussion

In the Southeast Asian countries of Vietnam, Cambodia, Laos and Thailand the primary malaria vectors are *An. dirus*, *An. minimus*, *An. maculatus*, and *An. sawadwongporni *with the relative importance of each varying, depending on the ecology of the area where transmission is occurring [15-22]. Other species are occasionally incriminated: *An. aconitus*, *An. jeyporiensis*, *An. philippinensis*, *An. nivipes*, *An. barbirostris*, and members of the *An. hyrcanus *group [16,19,21,22]. One feature common to all these species is that they are zoophilic and are found more often feeding on cattle and buffalo than on humans [19,23,24]; the only exception being *An. dirus*, which has consistently been shown to be an anthropophilic species and for this reason is the most dangerous vector of all the species mentioned above [16,18,19,21,25].

The low rate of malaria transmission found in the villages of Khe Ngang and Hang Chuon, two typical rural communities in north central Vietnam, was being maintained by a number of zoophilic species: *An. sinensis*, *An. harrisoni, An. maculatus, An. sawadwongporni*, *An. aconitus, An. peditaeniatus*, and *An. philippinensis*, This more or less reflects the findings of other workers in Southeast Asia [15,17,21,22]. Though in this study *An. sinensis *was found to play a relatively major role in malaria transmission and *An. dirus *was not found positive for CS antigen, though this might reflect the paucity of this species in the study area.

In Thailand and Laos, *An. sinensis *and other members of the Hyrcanus Group do not appear to play a role in malaria transmission; there are only two published records of them being possible vectors [21,25]. They are rarely recorded in large numbers [17,21-23,26,27], only in one village in Khammouane Province, Laos (on the border with Quang Binh Province, Vietnam) were members of this Group abundant, making up 44.2% of the catch off animal baits and 21.4% in HLC [19]. The reason for the low densities of *An. sinensis *recorded in surveys conducted in Thailand and Laos is unknown; in this study in Truong Xuan Commune, *An. sinensis *was strongly associated with rice growing, an activity that is ubiquitous throughout Thailand and Laos. *Anopheles sinensis *is more common to the north east of Vietnam throughout China and the Korean Peninsula where it is a major vector of malaria (along with other members of the Hyrcanus Group) [27-29]. In most surveys where Hyrcanus specimens have been collected there has been no attempt to separate the members of the Group [16,19,21,25]; this is due to major difficulties with the morphological identification of these species throughout their range in Southeast Asia and China [30,31]. It has been suggested that there is too much variation in many of the characters commonly used to assign affinities between members of this Group [31]. Certainly in this survey there were issues with separating species, with seven members of the Group being identified by morphology but this being resolved to three following molecular analysis.

Members of the Minimus Complex - *An. minimus *and *An. harrisoni *- have been found in Vietnam, though only *An. minimus *has been incriminated as a vector [15]. *Anopheles minimus *is thought to occur throughout the country while *An. harrisoni *is confined to the north (north of Quang Binh Province) [32], however the distribution of these two species is not well understood because their separation has been based on unreliable morphological wing characters [33]. Using DNA based identification technology [9], *An. harrisoni *has recently been recognised as occurring in central Vietnam with an apparent shift in dominance from *An. minimus *to *An. harrisoni *though the numbers collected and processed were small [34]. In this study, conducted in Quang Binh Province, north-central Vietnam over the period 2003-2007, *An. harrisoni *was the dominant species, accounting for 98.2% (1009/1028) of the *An. minimus *s.l. collected whereas *An. minimus *made up only 1.8% (19/1028). This study also incriminates for the first time *An. harrisoni *as a vector of malaria in Vietnam though as mentioned above this is a zoophilic species and its role in transmission is opportunistic.

The persistence of *P. falciparum *throughout the year will require continual human vector contact to be maintained. This is possible in Truong Xuan Commune due to both wet and dry seasons providing favourable conditions for the various vector species. Of the four most important vectors - *An. sinensis, An. harrisoni, An. maculatus *and *An. sawadwongporni *- *An. sinensis *occurred throughout the year but appeared to be more common in the dry season, *An. harrisoni *was also more common in the dry season, whereas *An. sawadwongporni *was more common in the wet season and *An. maculatus *was found throughout the year.

While important in the local context of malaria transmission in Truong Xuan Commune none of the species reported on here appear to be efficient vectors as indicated by the overall low malaria transmission rates and low sporozoite rates. The main reason being the zoophilic behaviour of these species of mosquitoes. The close temporal association of cattle and buffalo to human habitation was not for any intended zooprophylactic purpose but simply for security and ownership. Not all households possessed cattle or buffalo and if far enough removed from these blood sources most of the anopheline species recorded here would readily feed on humans as indicated by the HLC for *An. sinensis*, *An. aconitus*, *An. dirus*, *An. maculatus*, *An. sawadwongporni*, and *An. harrisoni*. In Pakistan, where the main malaria vectors show zoophilic tendencies and cattle are kept close to houses, the inhabitants of such households appear to be at a higher risk from malaria infections as opposed to households without cattle [35]. This is due to cattle attracting larger numbers of mosquitoes into the household area and thus increasing human vector contact. This does not appear to be the case in Vietnam, in this study paired collections off buffalo and humans separated by only 15 m buffalo attracted 97.4% (4489/4610) of the anophelines collected and only 2.6% (121/4610) were diverted to humans (Tables 9 and 10). Thus, while unintended, cattle and buffalo in Khe Ngang and Hang Chuon do play a zooprophylactic role.

Two behavioral characteristics were observed in the anophelines in Truong Xuan Commune that will reduce the impact of a malaria control strategies involving indoor residual spraying (IRS) and the use of long lasting insecticidal nets (LLIN). The early night feeding (6 pm - 9 pm) of *An. maculatus*, *An. sawadwongporni*, *An. dirus*, and *An. harrisoni *will allow avoidance of the insecticide through outdoor biting as village people are still active outdoors at these times and even if indoors prior to 9 pm are not likely to be protected by a bed net. Similar early night feeding behaviour of these species has been noted in other parts of their range [22,23,36,37]. In Thailand there is evidence that these four species all show a pronounced excito-repellency response to DDT and pyrethroids [38,39]. While a shift to early night biting may be a way of allowing these species obtaining a blood meal outdoors and thus avoiding the insecticide treated surfaces. However these species, with the exception of *An. dirus*, are essentially zoophilic and would be under little if any insecticidal pressure to change their behaviour. The second and more important issue is the large uncontrolled zoophilic component of the anopheline fauna that will enable anopheline numbers to remain high and at the same time provide sufficient numbers for the occasional feeding on humans to maintain malaria transmission. This zoophilic component will be unaffected by the use of IRS and LLIN. As the anopheline species collected from cattle and buffalo baits were found to rest on vegetation before and after feeding a possible control strategy would be the use of pyrethroids as a barrier spray on the vegetation directly surrounding the cattle and buffalo. Barrier spraying of vegetation has been used in various situations against a number of pest and vector species [40-42]. In Khe Ngang there are 48 houses and in Hang Chuon 26, but in Khe Ngang and Hang Chuon there are only four and five locations respectively where cattle and buffalo are kept at night. The area around the buffalo is approximately 4 m × 5 m; the area around the cattle varies with the number of cattle but the largest no bigger than 5 m × 10 m. The advantage of using barrier spraying in these villages in Truong Xuan is that mosquitoes are attracted and concentrated into a small area around the penned or tethered animals so only a small defined area would need to be sprayed and these are considerable less than the number of houses requiring spraying using conventional IRS. This would allow for multiple applications over a year to compensate for the poor residual effect of these insecticides applied in this manner [42,43]. This method of insecticide application would be more effective than the current permethrin IRS.

## Conclusion

In the rural areas of Quang Binh Province, north central Vietnam, where the forest has been largely cleared for agriculture and the primary malaria vector *An. dirus *is uncommon, malaria transmission was still maintained throughout the year by a number of secondary vectors - *An. sinensis*, *An. harrisoni, An. maculatus, An. sawadwongporni*, *An. aconitus, An. peditaeniatus*, and *An. philippinensis*. In this study the use of DNA-based identification methods made it possible to accurately incriminate the vector species and to assign relevant transmission parameters such as, larval ecology, biting behaviour, host preference, and seasonal abundance patterns to the appropriate species. The vector species were all predominantly zoophilic and were not particularly effective vectors, their differing preferences for oviposition sites over the year allowed for low level but persistent year round malaria transmission. The attractiveness of cattle and buffalo for these mosquitoes tends to concentrate mosquitoes into small and well defined areas within the village and as the host seeking mosquitoes rest on vegetation, in the immediate vicinity of the animal host both before and after feeding, there is an opportunity to implement barrier spraying. This may have a greater impact on anopheline densities and species longevity than the currently used control methods of IRS and LLIN.

## Competing interests

The authors declare that they have no competing interests.

## Authors' contributions

DMC and LQT coordinated and led the field and laboratory teams in the collection of specimens and the processing of specimens for species identification and circumsporozoite protein and contributed to the content of the manuscript. NWB provided intellectual input and support for the molecular identification of the anopheline species and contributed to content of the manuscript. NTHV carried out much of the processing of specimens for species identification and circumsporozoite protein. TLC, NVD, NXT and LNA all made substantial contributions to organising and supervising the collection of the field data. RDC designed the study, participated in the collection and processing of the specimens and drafted the manuscript. All authors have read and approved the final manuscript.

## References

[B1] Hung LQ de Vries PJ Giao PT Nam NV Binh TQ Chong MT Quoc NTTA Thanh TN Hung LN Kager PA Control of malaria: a successful experience from Viet Nam Bull World Health Organ 2002 80 660 665 12219158 PMC2567582

[B2] Barat LM Four malaria success stories: How malaria burden was successfully reduced in Brazil, Eritrea, India, and Vietnam Am J Trop Med Hyg 2006 74 12 16 16407339

[B3] Morrow M Nguyen QA Caruana S Biggs BA Doan NH Nong TT Pathways to malaria persistence in remote central Vietnam: a mixed-method study of health care and the community BMC Public Health 2009 9 85 10.1186/1471-2458-9-85 19309519 PMC2666724

[B4] Erhart A Thang ND Hung NQ Toi LV Hung LX Tuy TQ Cong LD Speybroeck N Coosemans M D'alessandro U Forest malaria in Vietnam: a challenge for control Am J Trop Med Hyg 2004 70 110 118 14993619

[B5] Erhart A Thang ND Ky PV Tinh TT Overmeir CV Speybroeck N Obsomer V Hung LX Thuan LK Coosemans M D'alessandro U Epidemiology of forest malaria in central Vietnam: a large scale cross-sectional survey Malar J 2005 4 58 10.1186/1475-2875-4-58 16336671 PMC1325238

[B6] Sang NH Dung NV Thanh NX Trung TN Co TV Cooper RD Forest malaria in central Vietnam Am J Trop Med Hyg 2008 79 653 654 18981498

[B7] Walton C Handley JM Kuvangkadilok C Collin FH Harbach RE Baimai V Butlin RK Identification of five species of the *Anopheles dirus *complex from Thailand using allele-specific polymerase chain reaction Med Vet Entomol 1999 13 24 32 10.1046/j.1365-2915.1999.00142.x 10194746

[B8] Manguin S Kengne P Sonnier L Harbach RE Baimai V Trung HD Coosemans M SCAR markers and multiplex PCR-based identification of isomorphic species in the *Anopheles dirus *complex in Southeast Asia Med Vet Entomol 2002 16 46 54 10.1046/j.0269-283x.2002.00344.x 11963981

[B9] Van Bortel W Trung HD Roelants P Harbach RE Backeljau T Coosemans M Molecular identification of *Anopheles minimus *s.l. beyond distinguishing the members of the species complex Insect Mol Biol 2000 9 335 340 10.1046/j.1365-2583.2000.00192.x 10886418

[B10] Walton C Somboon P O'Loughlin SM Zhang S Harbach RE Linton YM Chen B Nolan K Duong S Fong MY Vythilingum I Mohammed ZD Trung HD Butlin RK Genetic diversity and molecular identification of mosquito species in the *Anopheles maculatus *group using the ITS2 region of rDNA Infect Genet Evol 2007 7 93 102 10.1016/j.meegid.2006.05.001 16782411

[B11] Walton C Somboon P Harbach RE Zhang S Weerasinghe I O'Loughlin SM Phompida S Sochantha T Tun-Lin W Chen B Butlin RK Molecular identification of mosquito species in the *Anopheles annularis *group in southern Asia Med Vet Entomol 2007 21 30 35 10.1111/j.1365-2915.2006.00660.x 17373944

[B12] Dusfour I Blondeau J Harbach RE Vythilingham I Baimai V Trung HD Sochanta T Bangs MJ Manguin S Polymerase chain reaction identification of three members of the *Anopheles sundaicus *(Diptera: Culicidae) complex, malaria vectors in Southeast Asia J Med Entomol 2007 44 723 731 10.1603/0022-2585(2007)44[723:PCRIOT]2.0.CO;2 17915501

[B13] Beebe NW Maung J van den Hurk AF Ellis JT Cooper RD Ribosomal DNA spacer genotypes of the *Anopheles bancroftii *group (Diptera: Culicidae) from Australia and Papua New Guinea Insect Mol Biol 2001 10 407 413 10.1046/j.0962-1075.2001.00278.x 11881804

[B14] Beebe NW Saul A Discrimination of all members of the *Anopheles punctulatus *complex by polymerase chain reaction-restriction fragment length polymorphism analysis Am J Trop Med Hyg 1995 53 478 481 7485705 10.4269/ajtmh.1995.53.478

[B15] Trung HD Van Bortel W Sochantha T Keokenchanh K Quang NT Cong LD Coosemans M Malaria transmission and major malaria vectors in different geographical areas of Southeast Asia Trop Med Int Health 2004 9 230 237 10.1046/j.1365-3156.2003.01179.x 15040560

[B16] Vythilingam I Phetsouvanh R Keokenchanh K Yengmala V Vanisaveth V Phompida S Hakim SL The prevalence of *Anopheles *(Diptera: Culicidae) mosquitoes in Sekong Province, Lao PDR in relation to malaria transmission Trop Med Int Health 2003 8 525 535 10.1046/j.1365-3156.2003.01052.x 12791058

[B17] Coleman RE Sithiprasasna R Kankaew P Kiattibut C Ratanawong S Khuntirat B Sattabongkot J Naturally occurring mixed infection of *Plasmodium vivax *VK210 and *P. vivax *VK247 in *Anopheles *mosquitoes (Diptera: Culicidae) in western Thailand J Med Entomol 2002 39 556 559 10.1603/0022-2585-39.3.556 12061456

[B18] Vythilingam I Sidavong B Chan ST Phonemixay T Vanisaveth V Sisoulad P Phetsouvanh R Hakim SL Phompida S Epidemiology of malaria in Attapeu Province, Lao PDR in relation to entomological parameters Trans R Soc Trop Med Hyg 2005 99 833 839 10.1016/j.trstmh.2005.06.012 16112154

[B19] Toma T Miyagi I Okazawa T Kobayashi J Saita S Tuzuki A Keomanila H Nambanya S Phompida S Uza M Takakura M Entomological surveys of the malaria in Khammouane Province, Lao PDR, in 1999 and 2000 Southeast Asian J Trop Med Public Health 2002 33 532 546 12693588

[B20] Chareonviriyaphap T Bangs MJ Ratanatham S Status of malaria in Thailand Southeast Asian J Trop Med Public Health 2000 31 225 237 11127318

[B21] Rattanarithikul R Konishi E Linthicum KJ Detection of *Plasmodium vivax *and *Plasmodium falciparum *circumsporozoite antigen in anopheline mosquitoes collected in southern Thailand Am J Trop Med Hyg 1996 54 114 121 8619432 10.4269/ajtmh.1996.54.114

[B22] Harbach RE Gingrich Pang LW Some entomological observations on malaria transmission in a remote village in northwestern Thailand J Am Mosq Control Assoc 1987 3 296 301 3333058

[B23] Rattanarithikul R Konishi E Linthicum KJ Observations on nocturnal biting activity and host preference of anophelines collected in southern Thailand J Am Mosq Control Assoc 1996 12 52 57 8723258

[B24] Muenworn V Sungvornyothin S Kongmee M Polsomboon S Bangs MJ Akathanakul P Tanasinchayakul S Prabaripai A Chareonviriyaphap T Biting activity and host preference of the malaria vectors *Anopheles maculatus *and *Anopheles sawadwongporni *(Diptera: Culicidae) in Thailand J Vector Ecol 2009 34 62 69 20836806 10.1111/j.1948-7134.2009.00008.x

[B25] Rosenberg R Andre RG Somchit L Highly efficient dry season transmission of malaria in Thailand Trans R Soc Trop Med Hyg 1990 84 22 28 10.1016/0035-9203(90)90367-N 2189240

[B26] Kobayashi J Somboon P Keomanila H Inthavongsa S Nambanya S Inthakone S Sato Y Miyagi I Malaria prevalence and a brief entomological survey in a village surrounded by rice fields in Khammouan province, Lao PDR Trop Med Int Hlth 2000 5 17 21 10.1046/j.1365-3156.2000.00516.x 10672201

[B27] Li Z Luo D Liu X Malaria characteristics and control in Henan, People's Republic of China Southeast Asian J Trop Med Public Health 1995 26 402 406

[B28] Dapeng L Deling L Renguo Y Peng L Xueguang H Aimin L Lei W Changyin G Shaowen Z Hongru H Leyuan S Alphamethrin-impregnated bed nets for malaria and mosquito control in China Trans R Soc Trop Med Hyg 1994 88 625 628 10.1016/0035-9203(94)90199-6 7886750

[B29] Coleman RE Kiattibut C Sattabongkot J Ryan J Burkett DA Kim HC Lee WJ Klein T Evaluation of Anopheline mosquitoes (Diptera:Culicidae) from the Republic of Korea for *Plasmodium vivax *circumsporozoite protein J Med Entomol 2002 39 244 247 10.1603/0022-2585-39.1.244 11931266

[B30] Reid JA The *Anopheles hyrcanus *Group in South-East Asia (Diptera: Culicidae) Bull Entomol Res 1953 44 5 76 10.1017/S0007485300022938

[B31] Harrison BA Southeast Asia Mosquito Project A new interpretation of affinities within the *Anopheles hyrcanus *Complex of Southeast Asia Mosq Syst 1972 4 73 83

[B32] Garros C Van Bortel W Trung HD Coosemans M Manguin S Review of the Minimus Complex of *Anopheles*, main malaria vector in Southeast Asia: from taxonomic issues to vector control strategies Trop Med Int Health 2006 11 102 114 10.1111/j.1365-3156.2005.01536.x 16398761

[B33] Cuong DM Van NTH Tao LQ Chau TL Anh LN Thanh NX Cooper RD Identification of *Anopheles minimus *complex and related species in Vietnam Southeast Asian J Trop Med Public Health 2008 38 827 831 19058576

[B34] Garros C Marchand RP Quang NT Hai NS Manguin S First record of *Anopheles minimus *C and significant decrease of *An. minimus *A in central Vietnam J Am Mosq Control Assoc 2005 21 139 143 10.2987/8756-971X(2005)21[139:FROAMC]2.0.CO;2 16033115

[B35] Bouma M Rowland M Failure of passive zooprophylaxis: cattle ownership in Pakistan is associated with a higher prevalence of malaria Trans R Soc Trop Med Hyg 1995 82 351 353 10.1016/0035-9203(95)90004-7 7570859

[B36] Chareonviriyaphap T Parbaripai A Bangs MJ Aum-Aung B Seasonal abundance and blood feeding activity of *Anopheles minimus *Theobald (Diptera: Culicidae) in Thailand J Med Entomol 2003 40 876 881 10.1603/0022-2585-40.6.876 14765665

[B37] Sungvornyothin S Kongmee M Muenvorn V Polsomboom S Bangs MJ Prabaripai A Tantakom S Chareonviriyaphap T Seasonal abundance and blood feeding activity of *Anopheles dirus *sensu lato in western Thailand J Am Mosq Control Assoc 2009 25 425 430 10.2987/09-5907.1 20099588

[B38] Chareonviriyaphap T Prabaripai A Bangs MJ Excito-repellency of deltamethrin on the malaria vectors, *Anopheles minimus, Anopheles dirus, Anopheles sawadwongporni*, and *Anopheles maculatus*, in Thailand J Am Mosq Control Assoc 2004 20 45 54 15088704

[B39] Muenworn V Akathanakul P Bangs MJ Prabaripai A Chareonviriyaphap T Insecticide-induced behavioral response in two populations of *Anopheles maculatus *and *Anopheles sawadwongporni*, malaria vectors in Thailand J Am Mosq Control Assoc 2006 22 689 698 10.2987/8756-971X(2006)22[689:IBRITP]2.0.CO;2 17304938

[B40] Britch SC Linthicum KJ Wynn WW Walker TW Farooq M Smith VL Robinson CA Lothrop BB Snelling M Gutierrez A Lothrop HD Evaluation of barrier treatments on native vegetation in a southern California desert habitat J Am Mosq Control Assoc 2009 25 184 193 10.2987/08-5830.1 19653501

[B41] Perich MJ Tidwell MA Dobson SE Sardelis MR Zaglul A Williams DC Barrier spraying to control the malaria vector *Anopheles albimanus*: laboratory and field evaluation in the Dominican Republic Med Vet Entomol 1993 7 363 368 10.1111/j.1365-2915.1993.tb00706.x 8268492

[B42] Amoo AOL Xue R Qualls WA Quinn BP Bernier UR Residual efficacy of field-applied permethrin, d-phenothrin, and resmethrin on plant foliage against adult mosquitoes J Am Mosq Control Assoc 2008 24 543 549 10.2987/08-5783.1 19181063

[B43] Anderson A Apperson CS Knake R Effectiveness of mist-blower applications of malathion and permethrin to foliage as barrier sprays for salt marsh mosquitoes J Am Mosq Control Assoc 1991 7 116 117 1675251

